# Social and Cultural Factors Affecting Maternal Health in Rural Gambia: An Exploratory Qualitative Study

**DOI:** 10.1371/journal.pone.0163653

**Published:** 2016-09-23

**Authors:** Mat Lowe, Duan-Rung Chen, Song-Lih Huang

**Affiliations:** 1 Institute of Health Policy and Management, College of Public Health, National Taiwan University, Taipei, Taiwan (R.O.C); 2 Institute of Health Behaviors and Community Sciences, College of Public Health, National Taiwan University, Taipei, Taiwan (R.O.C); 3 Department of Public Health, International Health Program, National Yang-Ming University, Taipei, Taiwan (R.O.C); University of Otago, NEW ZEALAND

## Abstract

**Background:**

The high rate of maternal mortality reported in The Gambia is influenced by many factors, such as difficulties in accessing quality healthcare and facilities. In addition, socio-cultural practices in rural areas may limit the resources available to pregnant women, resulting in adverse health consequences. The aim of this study is to depict the gender dynamics in a rural Gambian context by exploring the social and cultural factors affecting maternal health.

**Methods and Findings:**

Five focus group discussions that included 50 participants (aged 15–30 years, with at least one child) and six in-depth interviews with traditional birth attendants were conducted to explore perceptions of maternal health issues among rural women. The discussion was facilitated by guides focusing on issues such as how the women perceived their own physical health during pregnancy, difficulties in keeping themselves healthy, and health-related problems during pregnancy and delivery. The data resulting from the discussion was transcribed verbatim and investigated using a qualitative thematic analysis. In general, rural Gambian women did not enjoy privileges in their households when they were pregnant. The duties expected of them required pregnant women to endure heavy workloads, with limited opportunities for sick leave and almost nonexistent resources to access prenatal care. The division of labor between men and women in the household was such that women often engaged in non-remunerable field work with few economic resources, and their household duties during pregnancy were not alleviated by either their husbands or the other members of polygamous households. At the time of delivery, the decision to receive care by trained personnel was often beyond the women’s control, resulting in birth-related complications.

**Conclusions:**

Our findings suggest that despite women’s multiple roles in the household, their positions are quite unfavorable. The high maternal morbidity and mortality rate in The Gambia is related to practices associated with gender inequality.

## Introduction

The Gambia, like many other countries in sub-Saharan Africa, has long been overburdened with maternal health problems. With a population of approximately 1.8 million, the densely populated West African nation has been ranked among the African countries with the highest levels of maternal mortality [1]. The national maternal mortality ratio, which has fallen by 46% over the last 20 years, is estimated at 400 deaths per 100,000 live births [2].

Despite progress in increasing the use of antenatal care and access to health care facilities [3], only 57% of births are attended by a skilled birth attendant [2]. The vast majority of women deliver at home under the supervision of a traditional birth attendant [3], and only one in five women with obstetric emergencies reports to a medical facility for assistance; there is a gross unmet need for emergency obstetric care, especially in public facilities [4, 5]. A woman in The Gambia also has a 1 in 23 lifetime risk of dying from maternity-related causes, and more than 50% of maternal deaths occur among women under 35 years of age [6].

Many studies have revealed a number of causes of maternal mortality in The Gambia. These include restricted access to emergency obstetric care [1], substandard quality of referral care [7], hemorrhage and related conditions such as hypertension and anemia [8], and endemic diseases such as malaria during pregnancy [9].

A recent survey also examined several barriers to skilled birth attendance in The Gambia, showing that the most frequently reported barriers to giving birth in a healthcare facility were insufficient time to travel (75%) and lack of transportation (29%) [2]. Other possible barriers include lack of confidence in healthcare facilities, financial cost of healthcare, domestic workload, and traditional practices that include a preference for birthing at home under the supervision of a traditional birth attendant [10]. Another review identified high maternal age, household wealth, education, low parity and urban residence as factors that predict the use of maternity services [11].

Although many of these studies have identified problems affecting maternal health, their focus has mainly been on quantifiable obstetric causes of maternal death and structural barriers to formal healthcare delivery services. In addition, qualitative studies have also typically focused on access to emergency obstetric care with little attention to the dimensions of women’s position within household [12]. Thus, in this study an attempt is made to explore the influence of socio-cultural factors of gender roles and relations within the household affecting maternal health in rural Gambia.

### The theoretical perspective on gender roles and relations

Many studies in the vast field of gender and health research on women’s lack of agency in rural sub-Saharan Africa (and elsewhere in the global south) have suggested various ways in which intra-household relations may affect women’s health. Evidence from diverse settings suggests that power relations are influenced by constructs at the interpersonal and societal levels [13, 14]. The manifestations of power are also shaped and in turn affected by social and normative prescriptions related to gender [15–18].

Drawing on these conceptual insights, this study employed the intra-household bargaining power theory pioneered by Sen (1990) and Agarwal (1997). In the context of “bargaining” and gender relations within the household, Agarwal [19] observed that the nature of gender relations–relations of power between women and men–is not easy to understand in its full complexity and that the complexity arise not least from the fact that gender relations (like all social relations) embody both the material and ideological, but are also revealed in the division of labor and resources between men and women. Based on these premises, Agarwal [19] observed that previous models of the household have paid inadequate or no attention to some critical aspects of intra-household gender dynamics such as: what factors (especially qualitative ones) affect bargaining power? What is the role of social norms and social perceptions in the bargaining process and how might these factors themselves be bargained? Are women less motivated than men by self-interest and might this affect bargaining outcomes?

As a demonstration of the useful of the intra-household bargaining power and gender relations, this study premised that gender related socio-cultural factors impinge intra-household bargaining power and retard maternal health care utilization. The type of society (for example, patriarchic or traditional) a woman lives in and the gender norms and values within the society determine her status within the community and household, thereby inhibiting women access to health care [20]. The dynamics of the relationship between a woman and her partner can also influence access to and control over resources and decision on how to expend resources, which ultimately has implications for maternal health. Therefore, the aim of this study is to depict the gender dynamic in a rural Gambian context from the perspectives of women by exploring the social and cultural factors affecting maternal health. Our study findings contribute to the understanding of the gendered patterning embedded in interpersonal relations and have implications for reducing maternal mortality in resource poor settings.

### Literature review

Maternal health improvement has attracted global attention at the 1987 Safe Motherhood Conference held in Nairobi, Kenya. Since then, improving women’s health issues pertaining to pregnancy and delivery has become the centerpiece of national development efforts in developing countries. However, despite this significant stride, there is little evidence to prove that maternal mortality has declined significantly in African countries, including The Gambia [21]. Review of related studies throws more light on why African countries are held back on maternal mortality reduction.

A study of Cameroonian women reported that although women were generally worried about their health, the cultural background of gender roles blinded them from recognizing their right to maintaining good health [22]. These women considered the right to good health as contingent on fulfilling their purpose of taking care of and meeting the needs of “others” (such as husbands) at the expense of their own physical health and well-being. There is a religious and socio-cultural dimension for this consideration. In a study among the Hausa of Northern Nigeria, Afonja [23] found that the most important factors contributing to maternal deaths include an Islamic culture that undervalues women; a perceived social needs for women’s reproductive health capacities to be under strict male control and the practice of purdah (wife seclusion), which restrict women’s medical care; almost universal female illiteracy; marriage at an early age and pregnancy often occurring before maternal pelvic is complete and harmful traditional medical practices among others [24].

Dixon et al. [25] also found intra-household dynamics impact male/female enrolment in national health insurance in Ghana. In this case mothering often prevented women from enrolling. Similarly, another study in Mali found women’s own perceptions of their self-efficacy and the value of women in society as determinants for their preventive and health seeking behavior [18]. A study in Benin Republic also reported that factors like husbands’ approval and money for treatment had negative effects on maternal health seeking behavior [26]. This finding shows the lack of decision-making autonomy and economic independence of women.

Women’s decision-making autonomy may be explained in relation to their lack of education and limited influence over material resources [12]. Women’s land rights in particular have been a major focus of empowerment efforts [27], yet only in a few countries do women constitute even one-quarter of the landowners [28]. This gender disparity in land ownership impacts the economic status of women and further perpetuates a high level of dependency on their husbands, leading to male dominance [24]. Adding another voice to this discussion, Hansford et al. [29] observed that although women may have access to land or retain control over their own income, many have no influence on the use of their husband’s income. This may mean that women are particularly unlikely to make independent decisions about how money should be spent on high-expenditure items, including healthcare services.

The dynamics of the relationship between a woman and her husband can also determine a woman’s access to healthcare and can lead to varying levels of partner-controlling behaviors, such as gender-based violence [30], which studies have shown is associated with poor maternal health. A survey among antenatal care clinic attendees in The Gambia in 2011 revealed (61.8%) prevalence of intimate partner violence among study subjects, with 12% requiring medical care and 3% prevented from seeking healthcare as a result of such violence [31]. Another study in Matlab, Bangladesh, found that homicides and suicides that were motivated by stigma over unwanted pregnancy, beatings or dowries accounted for 6% of all maternal deaths in 1986 [32].

Other traditional practices based on patriarchal beliefs also affect women’s psychological and physical health [22]. Polygamy, a common marital practice in many African countries, is deeply engrained in Gambian culture, where multiple partners are considered part of Gambian masculinity [33]. This practice has resulted in men abandoning older wives for younger ones, leading to a vicious cycle of abuse toward women [33]. Many studies have shown that women in polygamous unions, especially senior wives, may suffer from more psychological disorders, as well as more familial and economic problems, compared to their counterparts in monogamous marriages [34, 35].

Taken together, these issues highlight the consequences of women’s living conditions in relation to their disadvantaged experiences, which are also connected to the gendered effects of socio-cultural practices affecting maternal health. This underscores the significance of this study, whose objective was to depict the gender dynamic in a rural Gambian context from the perspectives of women by exploring the social and cultural factors affecting maternal health issues pertaining to pregnancy and delivery. A key focus of the study is on household dynamics.

## Materials and Methods

### Design

This study was based on a qualitative, exploratory research design and used focus group discussions and in-depth interviews as its primary data collection techniques.

### Setting

We conducted focus group discussions and in-depth interviews in five rural communities (Makka Farafenni, Kerr Ardo, Kerr Gumbo, Mballow Omar and Bakindik) in the North Bank Region of The Gambia, where farming and animal rearing remain the mainstays of the local economy. The three main ethnic groups (Mandinka, Fula and Wolof) in the country reside in these communities. Our choice of these five rural communities was based on their long-standing history as locations of research intervention and continuous demographic surveillance [36]. In 1987, they were among the primary research sites for the field trial of the sisterhood method in The Gambia, which indicated a lifetime risk of maternal mortality of 0.0584, or 1 in 17 [37]. The sisterhood method is an indirect technique for deriving population-based estimates of maternal mortality [37].

It is also common to find women collaborating with research organizations in these five rural communities. This study partnered with the Agency for the Development of Women and Children (ADWAC) in order to secure entry points and easy access to the study communities and participants.

### Data collection

The data collection involved women residing in the five rural communities (Makka Farafenni, Kerr Ardo, Kerr Gumbo, Mballow Omar and Bakindik). Participants in the study were recruited from women’s groups existing in the study communities. They were contacted by ADWAC field coordinators, who briefed them about the objective of the study and requested for their assistance in identifying potential subjects. Fifty eligible participants were then identified. The sample inclusion criteria include women of childbearing age (15–30 years) with at least one child. It was assumed that participants meeting these criteria would be able to provide current information about maternal health issues because they had recently entered their reproductive years or had given birth relatively recently. Therefore, they would be more likely to recall pregnancy and other related complications. These groups of women may also be more exposed to maternal health research or more informed about issues affecting their health and pregnancies than other local women who do not belong to any women’s groups.

A total of five focus group discussions (FGDs) that included fifty participants and six in-depth interviews (IDIs) were conducted. The FGDs were held in the morning before the women started their work in the field, and typically lasted for 70–90 minutes. Each focus group discussion was limited to ten participants for ease of management and was held in either the village health post or community development center to avoid noise and distraction.

Following the introduction and informed consent routines, participants were asked to discuss the difficulties they face in keeping themselves healthy and health-related problems they had experienced during pregnancy and delivery. Based on the issues they raised, the researcher prompted them to describe their situations, how relevant decisions were made within their households, and how they managed to cope with difficulties. This discussion was supplemented by interactive questions about such topics as obtaining prenatal care, travel arrangements at the time of delivery and division of labor when they were pregnant. An interview guide mutually developed and agreed upon by the three authors (ML, DR and SLH) was used to facilitate the discussion. The interview guide was developed based on literature review of previous studies on women’s perceptions of maternal health issues [38], and on social and cultural barriers of maternity care [39, 40]. The interview guide was pilot tested with participants that have similar inclusion criteria as those that participated in the study and was ethically approved long before primary data was being collected and no change was made following ethical approval. It was designed to be open ended and contained specific questions, such as the following: “What are the obstacles pregnant women in your community face in seeking and receiving care?”; “Do any of you travel to a nearby place in anticipation of delivery?”; “Was this visit a burden to your family or yourself?”; “How were transportation arrangements made for you?”; “Who made the decision for you to seek care?”; and “Why was this person very important in your decision to seek care?”.

In-depth interviews (IDIs) were also conducted with six traditional birth attendants (TBAs) to obtain in-depth information about maternal health issues in their communities. The traditional birth attendants were crucial to the data collection because they are considered part of the Gambian healthcare system [41]. TBAs have been practicing in the communities even before The Gambia adopted the primary healthcare program in 1978. They work (often in conjunction with village health workers), make referrals and attend all forms of illness in the village.

The in-depth interviews with TBAs were conducted to enrich data from the FGDs and to facilitate data triangulation [42]. The data collection was limited to five focus group discussions and six in-depth interviews because of the complexity of the data, which were time consuming in terms of collecting, and because the data aimed to provide rich insights to understand social phenomenon rather than statistical information. The number of focus group discussions and in-depth interviews were decided upon beforehand by the three authors (ML, DR, and SLH). The rationale for the use of these qualitative methods was to draw upon participants’ experiences and reactions in a way which would not be feasible using other methods [43].

The data collection process for both the FGDs and IDIs was based on the principle of saturation [44, 45] and was conducted during August and September 2012, with two research assistants recording all the discussions. The research assistants were a female nurse and a male community development worker who had mastery of the two local languages (Mandinka and Wolof) and had experience in data collection.

### Data analysis

For the more rigorous part of the data analysis, all sound recording files from the FGDs were uploaded to a computer and password protected; they were then translated from the vernacular language into English and transcribed verbatim by (ML) and the research assistants. ML played and listened to the audio recorder several times in full before transcription began. After transcription, familiarity with the data was developed through many readings of the entire transcripts; to obtain a sense of totality; significant statements were highlighted and extracted. Meanings of significant statements and sentences that bear similar attributes were then labeled and coded by the three authors (ML, DR and SLH). Open coding with paper and pen was used at this stage, in which different parts of the text that contained significant statements were marked with appropriate labels and coded for further analysis. Coding and analysis of all recordings were subjected to manual thematic analysis [46].

During analysis of the data, the study used common properties to group descriptions of similar situations or ideas into key concepts. Concepts with common properties were then classified based on the study objective and the data collected. The strategy used for coding was constructed code, which uses either coded data from in vivo codes (created by the researcher) or from academic terms. We created our own codes based on the study objective and question guide.

After coding the data, a table was created. ML copied and pasted the coded data into the table in order to group the themes to ensure that no code has been missed and that the described and named themes provide a clear picture of what each theme was about [46]. The themes comprised at least two quotes followed by summary accounts or comments from the TBAs. We cross-examined the interviews with the observations of the TBAs to determine whether the experiences reported by the participants were also observed by the TBAs. Particular attention was paid to how many participants shared a certain idea in some of the quotations (using expressions such as “the majority”, “a few” and “several participants”). We also added more “context” around some of the verbatim data, using additional data regarding the participant's profile (such as age, number of children and marital status). Participants’ quotes were reported directly as they were spoken, without editing the grammar, to avoid losing meaning [47].

### Trustworthiness

The study employed member checks and prolonged engagement with participants and research assistants to strengthen the credibility of the accounts [48, 49]. For the member checks, participants who were willing to stay following each focus group discussion were asked to listen to the sound recordings and if they agreed with what had been said in the sound recordings. This was supplemented by prolonged engagement with the research assistants in the form of peer debriefing throughout the general data processing to ensure that the data were processed and presented succinctly. The prolonged engagement with the research assistants was facilitated by an observational protocol that was designed for use by research assistants during FGDs. The observational protocol contained a reflection component, which was built in to facilitate peer debriefings after focus group discussions and to better prepare for ensuing sessions [48]. Local words or dialects referring to particular illnesses or perceptions were also translated into English by two bilingual speakers external to the research.

### Ethics

The study was approved by The Gambia Government/Medical Research Council (MRC) Joint Ethics Committee. Before the start of any discussion participants were informed about the purpose of the study and all what was required of them as respondents, such as the reason for them to stay to the end of the discussion. They were told that they reserve all the rights to participate and not to participate. The main researcher and first author (ML) informed them that all the discussion will be recorded on an audiocassette for his own use, to which all the participants consented to. This method of obtaining informed consent is common in The Gambia and was approved by the Ethics Committee.

Verbal informed consent was used given the illiteracy rate in The Gambia, especially among women who formed the majority of the population. This posed a challenge to the data collection procedures that written informed consent was not possible. Sugarman et al. [50] also suggested that thumbprints might be considered to be an appropriate means to obtaining individual informed consent but explained that it too is closely linked to legal commitment and might frighten potential research participants.

Since the main researcher and one of the research assistants were men, permission was also taken from husbands of all the women who participated in the discussions because these men can decide whether their women should talk to other men or not.

## Results

The social and cultural factors affecting maternal health in rural Gambia are multidimensional and interlinked, but include the interplay of the following factors: (1) pregnant women’s heavy workload, (2) division of labor within the household, (3) women’s unfavorable position in the household and (4) limited access to and utilization of health care.

### Pregnant women’s heavy workload

When asked what they thought was detrimental to their health during pregnancy, the participants agreeably reported that their daily activities were too strenuous and that they could not get sufficient rest even as they approached their delivery date. They worked from the time they woke up until the time they went to sleep and were physically and emotionally exhausted by their field work, which was followed by house chores. The participants often said that they worked longer hours than their husbands, who were not necessarily fully occupied by their tasks.

A woman described her first delivery as follows: “While working on the farm; picking groundnut, I started experiencing abdominal pain. Then, I decided to return home but before I got home; I had already started seeing blood. And as soon as I reached home, the TBA came and that’s where I delivered. It was my first born baby”.

In many ways, it was this same multitude of household tasks that prevented women from seeking health care in the earlier stages of their pregnancies. In one focus group discussion, a polygamous woman, age 27 with five children, stated: “Here in this village, we only go to the hospital when we are in labor. We normally don’t travel to other places in preparation for delivery because we are usually constrained by our work, and it is difficult to stop working because your co-wives or people in the household may think differently as soon as you excuse yourself from normal household chores or from works in the farms”.

The physicality of women’s domestic and emotional work burden was also reaffirmed by a TBA, who related it to her own daughter’s ordeal. She echoed: “My last born, she is married; during her last delivery, she spent all day long working on the farm until when she returned from the farm that she started complaining of abdominal pain and suddenly delivered. Can you imagine? She is the only one doing the household work at her home”.

The TBA further noted: “One of the heaviest domestic chores performed by women in this community is pounding grains like coos or maize. You can be in your ninth month of pregnancy, but still you have to pound grains like coos or maize and go to the farm every day during farming season. Sometimes we send women to the hospital, and at the hospital, it is found out that she either has no water [dehydrated] or insufficient blood [anemic]. It is very common in this village and constitutes a serious health problem for women”.

Numerous testimonies like these indicated some of the difficulties encountered by pregnant women regarding workload and division of labor within the household. These issues may be complicated in polygamous households, where there often is rivalry among co-wives.

### Division of labor within the household

Despite the fact that some women had some knowledge of the maternal health risks involved in carrying out heavy domestic chores late in pregnancy, they continued to perform them. One possible reason was that they lacked support from their husbands. One participant had this to say: “When you are few months pregnant, it is not bad to do certain domestic chores, but at some point when you are six months or more, there is certain work like carrying buckets of water from the well to your home, fetching or splitting firewood and so on that you shouldn’t do. But there’s no alternative; if you don’t have anyone to do it for you, you must do it yourself no matter what”.

Another reason for the continuation of strenuous labor during pregnancy is that women feel obligated to care for themselves and their families. A woman with some years of education expressed sympathetically, that she observed some domestic chores performed by pregnant women–such as cooking while inhaling smoke from firewood, fetching and carrying water from long distances and picking groundnuts–as exposing them to major health risks: “In this community, women are exposed to such a heavy physical and emotional work burden, which I believe really endangers our pregnancies and lives as women. But we have to do them to feed our husbands and families”.

Women’s daily activities, such as picking groundnuts from the farms, are necessary for their own survival and self-sufficiency, as explained by one participant: “And if the husband doesn’t help his wife, the woman must work so that she can have something to take care of herself. And even if your pregnancy advances, you have to work in order to feed yourself and satisfy your basic needs and that of your family”.

A TBA added that women considered the consequences of their actions from the perspectives of others and not just of themselves, and they regarded the wellbeing and health of their families as more important than their own. She noted: “I delivered a woman yesterday who spent all day long working on the farm, and it was after she returned from work that she delivered. Women in this community work a lot. We work a lot because of our families”.

Men’s work is less demanding than women’s, and people tend to follow the traditional male-female division of labor, leaving pregnant women with their heavy daily workload. One woman described the situation vividly: “Some men during the dry season will be sitting at the ‘penchi’ (a common meeting place usually for men and boys). They expect you to go to the farm to pick groundnut, and at the end of the day, they want you to prepare for them a delicious food from the little money you got from your groundnut sale.”

Another participant added: “They will be relaxing at the ‘penchi’ when you are busy picking groundnut on the field, and upon your return, you have to take care of the kids, wash dishes, cook lunch and do all the household work while they are still relaxing. They are busy only during the raining season, but for us, we don’t have any off season. We are busy working both dry and rainy seasons”.

Field observations confirmed women’s stories about the seasonality of and contrast between the work of men and women. On several occasions during the course of data collection, men were observed in groups from morning until mid-day, and at mid-day they would move to a shadier place in order to avoid the sunlight and continue chatting. Meanwhile, women were carrying buckets of water, going to and coming from the farms. These observations suggest that women need to work daily and perhaps have limited cash income from their farm output.

The majority of participants explained that although they work alongside their husbands on the farms they have little or no economic return from their farm work. They reported that the majority of the income belonged to the husband. Women do not receive cash other than what is meant for daily expenses, and because of this limited income they must work extremely hard after harvest to obtain personal funds, including those for medical expenses. This participant explained as follows: “We are obliged to help our husbands on the farm until it is harvesting season, and after harvest, we can find the remaining groundnut in the ground that was left during the first harvesting for our own personal savings”.

Another participant added: “It is these funds that we [women] use to pay for medical and other related expenses, like transport fare, when your husband refused to provide you with money for medical check-ups”.

From the above statements it is clear that the work women perform is necessary to meet their husbands’ expectations as well as for the maintenance of themselves and their families. The contrast with regard to seasonality of work between men and women also shows that women are limited in their resources, including income, time for themselves, and opportunities to seek care. Women must work hard to fulfill their duties as wives and mothers in their families as well as to be economically self-sufficient.

### Women’s unfavorable position in the household

Despite their multiple roles in contributing to the family, women’s positions in the household were sometimes unfavorable. For example, they did not have the power or liberty to stop their work even as illnesses and complications arose during pregnancy. Many participants reported that they are not at liberty to stop their daily activities without the advice of a medical practitioner. A number of participants reported that their own words were not enough to obtain a respite from work. In one of the focus group discussions, when asked if women are relieved from normal household chores at any particular time, a married woman with three children, age 30, simply laughed aloud and explained: “We really work very hard. In this village, there is no free time even for pregnant women. You can only be relieved from domestic chores with the advice of health personnel. And that has to be in the presence of one of your close relatives or neighbors who will bear witness to you if there is any doubt when you reached home before you can stop working”.

This lack of flexibility in job arrangements within the household may be because the household head does not fully understand women’s issues. The majority of the participants explained that it was difficult to discuss pregnancy issues with their husbands. One woman with three children said: “I feel ashamed to discuss pregnancy and sexually related issues with my husband because I don’t believe it is traditionally accepted. It is seen as a taboo here in this village”. As a result, the husband was usually not involved in resolving disputes about work assignments among co-wives, especially when such disputes were related to pregnancy.

Another manifestation of the unfavorable position of women was their lack of control over material resources in the household. In the communities where this study was conducted, properties such as land and local means of transport (like horse-carts) are concentrated in the hands of men who are regarded as household heads and have authority over their use. Men have access to land for both cash cropping and subsistence farming. As explained above, the majority of income from farming belongs to the husbands. Women’s access to and control over resources presents practical challenges, and even though women strive to take care of everyone in the household, their needs are not necessarily taken seriously. A woman with three children had this to say with regard to antenatal examination: “Anytime I ask my husband money for transport fare to the clinic, he will complain that he doesn’t have money, but at the same time, you will see him spending money on other things. I either have to borrow or use my own saving if I have it. Or else ask my immediate family members, my brother, to help me”.

Another woman stated: “Whenever I want to go to the antenatal care, if I ask my husband for money for transport, his complaint is that he has no money, and if I insisted, it resulted in problems. He gets annoyed easily and will start to insult me”. Several participants said that asking for money might even bring about incidents of domestic violence.

These statements show that as female members of the household, pregnant women do not enjoy privileges such as taking sick leave from ordinary work or receiving assistance in having antenatal check-ups. Part of the problem is their lack of economic independence, but other reasons may contribute to their unfavorable position within the household.

### Limited access to and utilization of healthcare

Women’s work burden is also linked to the limited time they have available to access and use healthcare. The time they spend on performing their daily activities prevents them from travelling to nearby healthcare facilities in anticipation of delivery or delivery related complications, as explained by this participant: “Here in this village, we only go to the hospital at the onset of labor. We normally don’t travel to other places in preparation for delivery because we are usually constrained by our work. We have a lot of work to do at home”.

The limited time women have for themselves, as a result of their excessive workload, was one reason for their inability to access emergency obstetric care at the onset of labor. It also explains their preference for birthing at home under the supervision of a traditional birth attendant. In one of the FGDs, a woman with four children explains the situation as follows: “If you don’t have transport, you just deliver at home with the TBA because she is also trained to assist women”. This issue resulted in situations where women had limited access to institutionalized care for delivery purposes and did not use such care; instead, they consulted TBAs more routinely.

These factors together might at least partly explain the high risks associated with pregnancy and delivery. Maternal health issues will continue to be important in this part of rural Gambia and beyond.

## Discussion

Women in The Gambia faced major challenges accessing maternal health care for the entire duration of pregnancy; these challenges were particularly threatening during delivery. This study explores some of the social and cultural factors associated with women’s health during pregnancy and delivery. The study employed a qualitative approach involving focus group discussions and in-depth interviews with participants who were purposively recruited from women’s groups in the study communities. These groups of women provided rich insights into maternal health issues. Although we do not have concrete data, it is likely that these women are more informed and open to talking about maternal health issues than women who had not been exposed to maternal health discussions in general. However, we believe that the participants faithfully reflected the ways in which women interpreted and responded to health issues associated with pregnancy and delivery, as well as the within-household mechanisms affecting their health behavior. In particular, women’s limited opportunities for sick leave, the biased distribution of resources, their work burden, and limited access to and utilization of healthcare are among the most salient problems.

In many focus group discussions, the physical and emotional work burden and the lack of rest throughout the duration of pregnancy was the first issue to be raised by participants when they were asked about health issues associated with pregnancy. Previous studies have also indicated a worsening of women’s health in rural sub-Saharan Africa in relation to continued intense activity throughout pregnancy [51, 52]. Other studies have also found an association between specific tasks (like standing for long hours and climbing stairs) and difficult birth and miscarriage [53]. Our findings show that some reasons for the necessity of working in the field until the day of delivery included the following: first, this is common practice and is accepted by both men and women without question. Second, women generally do not have cash incomes, let alone savings, and need to work daily. Third, there is generally not a mechanism of work sharing within the household, either between the husband and wife or among co-wives in a polygamous family. The underlying situation is that women are not economically independent, and they have limited control over material and social resources. These same factors also limited women’s freedom to use the horse-cart or pay for other means of transportation to obtain healthcare services. It is also evident that the responsibility of paying for healthcare expenses rests heavily on women.

The unfavorable position occupied by women in this study is also manifested in their rights to land ownership. While there is an association between land rights and women’s empowerment [27], this study found that the land tenure system is biased against women. Land was, in most cases, reported to be owned and almost entirely controlled by men. Women reported limited control and utilization of land for income generating activities and/or to raise money for maternal healthcare expenditures. Therefore, it seems that land ownership and the opportunity for cash cropping may be at the root of improving maternal health problems.

This policy has wide health implications aside from maternal healthcare utilization, as mothers who own land are less likely to have severely underweight children [27]. The literature has also suggested that availability of and access to land constitute one form of autonomy for women; studies have clearly demonstrated that this has a strong effect on reproductive health outcomes [54].

Women’s workloads became more complicated in polygamous households, where there is often rivalry among co-wives. Polygamy, a common marital structure in many African societies including The Gambia, mediates important intra-household relationships by requiring both competition and pragmatic cooperation among co-wives [55, 56]. For this reason, women could not release themselves from farm work even as pregnancy complications arose. Polygamous women are also less likely to obtain treatment for which a monetary fee is required [55]. This confirms our findings that women need to work exceedingly hard to save money for their medical and other related expenses like transport fares, which are unlikely to be provided by polygamous husbands.

Despite their productive and reproductive role, such as in their participation in farm work and child rearing, women reported limited opportunities for sick leave. Several participants reported that they do not have the power to release themselves from domestic chores or work on the farm even as pregnancy complications arise. The decision to take sick leave or release themselves from domestic chores or work on the farm has to be approved by the husband in most cases, due to the patriarchic nature of the household. This gives men the right to make decisions that affect members of the family, such as allocation of resources to education, healthcare and high expenditure items. Our finding is in agreement with other studies showing that giving women decision-making powers in partnership with their spouses could raise the rates of delivery at healthcare facilities [57].

Regarding work sharing, our study observed that men have not taken responsibility for household chores, probably because they simply cannot see what needs to be done in the household due to the way they are acculturated, or perhaps because they do not see the need or do not believe that household chores warrant their efforts compared to other competing social responsibilities. Several participants in the focus group discussions revealed that their husbands are usually not aware of the pregnancy until it reaches a rather late stage, indicating limited communication between spouses. In addition, husbands’ roles are also shaped and restricted by cultural practices: according to our interviews, men who help with household chores may be subjected to mockery in the neighborhood. Social stigma against men who engage in household chores, in addition to the gendered and generational division of labor and the traditional approaches used to implement reproductive health programs, has also been found to be a prominent social barrier that inhibits male participation in maternal health [34, 58–60].

The participants also reported limited choice for seeking care and this was one reason for their heavy reliance on TBAs, who are considered to be more secretive and friendly than professional healthcare workers [41]. Taken together, these issues highlight some of the social and cultural factors affecting maternal health in rural Gambia. The qualitative data presented in the paper provide insights into the structure of gender relations, including some important elements for understanding the gendered patterning embedded in interpersonal relations. The data also elucidate the mechanisms by which health consequences are produced in rural Gambia. The study, however, is limited in its research design, which must be considered when interpreting the findings. First, it was undertaken in the catchment of North Bank Region, which has been under demographic surveillance for over 30 years [61]. Therefore, the potential implication of respondents being much more comfortable with researchers and perhaps anticipating the types of things researchers are interested in hearing is acknowledged. Second, participants in the study were recruited from women’s groups existing in the study communities, which may have biased the study sample. We, however, have aimed to minimize dominant respondent bias from women who were seemingly more informed about maternal health issues in the group discussions by asking probing questions of women who were less active in the FGDs. This was done to ensure that each participant was given the opportunity to participate in the discussions. Third, since the main researcher and one of the research assistants were men and needed to take permissions from husbands, the social and cultural positions of the researchers and research participants may have potential limitations on the study findings [62]. However, most women were opened to talk about maternal health issues because there are male practicing healthcare workers (including midwives, nurses and public health officers) in The Gambia and women talk to them about maternal health issues. Finally, although the study has touched on some important factors affecting maternal health, the findings are by no means all the social and cultural factors that impact maternal health and maternal care seeking in rural Gambia.

Regarding future research, this study indicates the need for “micro-demographic studies” that incorporate anthropological perspectives to assess the wide range of factors that affect maternal health in societies with different subsistence practices and a specific gendered social organization.
